# Combination of C-reactive protein and fibrinogen-to-albumin ratio as a novel predictor of all-cause mortality in heart failure patients

**DOI:** 10.1515/med-2024-1045

**Published:** 2024-11-18

**Authors:** Sirui Yang, Hongyan Cai, Zhao Hu, Wei Huang, Qin Fu, Ping Xia, Wenyi Gu, Tao Shi, Fazhi Yang, Lixing Chen

**Affiliations:** Department of Cardiology, Kunming Medical University First Affiliated Hospital, Kunming, Yunnan, China; Department of Geriatrics, The Third People’s Hospital of Yunnan Province, Kunming, Yunnan, China; Cadre Rehabilitation Department, Kunming Medical University First Affiliated Hospital, Kunming, Yunnan, China

**Keywords:** C-reactive protein, fibrinogen/albumin ratio, heart failure, inflammation, prognosis

## Abstract

Heart failure (HF) is a common cardiovascular disease that is related to systemic inflammation. This study aimed to assess the role of C-reactive protein (CRP) combined with fibrinogen-to-albumin ratio (C-FAR) on the prognosis of all-cause mortality in different types of HF. A total of 1,221 hospitalized HF patients from the First Affiliated Hospital of Kunming Medical University between January 2017 and October 2021 were retrospectively analyzed. Patients were categorized into a low C-FAR group (C-FAR < 0.69) and a high C-FAR group (C-FAR ≥ 0.69) according to the median C-FAR value. We used Kaplan–Meier plots, restricted cubic spline regression, Cox survival analyses, and time-dependent receiver operating characteristic (ROC) analyses to evaluate the prognostic role of C-FAR on all-cause mortality in different types of HF. After excluding patients lost to follow-up and those with missing data, we ultimately included 1,196 patients with HF. The Kaplan–Meier plots showed that HF patients with high C-FAR levels had a significantly greater risk of all-cause mortality. In all four Cox proportional risk models, C-FAR was an independent predictor of all-cause mortality. Based on the ROC curve, the area under the curve (AUC) for C-FAR was greater than the AUC for Lg BNP. In the subgroup analyses, patients had the highest risk of all-cause mortality when FAR ≥ 0.091 and CRP ≥ 7.470. Regardless of the type of HF, C-FAR can be a good predictor of prognosis for all-cause mortality in HF patients, and patients with high C-FAR had a significantly increased risk of death compared to those with low C-FAR.

## 1 Introduction

Heart failure (HF) remains a rising global epidemic and the fastest-growing cardiovascular disease (CVD) in the world [1]. Despite improved survival, patients with HF are still at risk of substantial mortality or recurrent decompensation requiring hospitalization [2]. The 2021 ESC HF Guidelines state that patients with HF can be divided into three categories according to left ventricular ejection fraction (LVEF): HF with reduced ejection fraction (HFrEF) is defined as an LVEF <40%; HF with mildly reduced ejection fraction (HFmrEF) is defined as an LVEF of 40–49%; and HF with preserved ejection fraction (HFpEF) is defined as an LVEF ≥50%. There are also differences in risk factors, pathophysiology, and treatments among different types of HF [3]. HF is characterized by chronic inflammation, both locally in the heart and in the circulation, so chronic systemic inflammation is associated with an increased risk of HF. More precisely, the high plasma concentrations of several proinflammatory cytokines are closely related to disease progression and poor prognosis [4,5].

C-reactive protein (CRP), an acute-phase reactant predominantly released from hepatocytes, is a nonspecific inflammatory marker clearly associated with adverse CVD outcomes [6]. Previous observational studies reported that CRP, as a representative biomarker of systemic inflammation, can predict the development and prognosis of HF [7,8,9].

Another inflammatory marker, fibrinogen (FIB), is an important determinant of blood viscosity and platelet aggregation and is also considered to be related to HF risk [10]. Some studies have confirmed the relationship between low serum albumin (ALB) and an increase in CVD incidence and mortality [11,12,13]. The fibrinogen-to-ALB ratio (FAR), which comprises these two indicators, is independently associated with the presence and severity of coronary artery disease and may serve as a potential prognostic indicator for patients with CVD [14,15]. A study by Rong Huang et al. demonstrated that FAR was independently associated with adverse prognosis in patients with acute decompensated HF in diabetes mellitus [16]. Xu et al. found that the FAR is an independent prognostic risk factor for 90-day and 1-year all-cause mortality among HF patients [17].

Several studies have shown that FAR and CRP are associated with the prognosis of HF patients [16,18,19], so we speculate that FAR combined with CRP may also affect the prognosis of patients with HF. The aim of our study was to evaluate the clinical prognostic impact of FAR combined with CRP in worsening HF patients with different ejection fractions.

## 2 Materials and methods

### 2.1 Study population

A retrospective analysis of 1,221 consecutive patients with acute exacerbation of chronic HF admitted to the First Affiliated Hospital of Kunming Medical University from January 2017 to October 2021 was carried out. The enrolled patients with HF had a New York Heart Association (NYHA) functional class of III or IV and brain natriuretic peptide (BNP) levels of at least 500 pg/mL on admission. After excluding patients with missing data (blood test results, echocardiographic data), other serious illnesses (malignancy, infections, or severe renal or hepatic impairment), and those who were lost to follow-up, 1,196 patients remained and were included in this study.

### 2.2 Data collection and definitions

Patient demographics (including age and sex), clinical history, anthropometric data, laboratory test results, echocardiographic data, and information on medication use during hospitalization were collected at admission. Some peripheral venous blood samples were collected on admission for routine blood tests, while some were collected after overnight fasting (>8 h), and the different variables were measured in the laboratory. FIB, ALB, CRP, D-dimer, troponin, creatine kinase-MB, BNP, hemoglobin, uric acid (UA), creatinine, white blood cell (WBC) count, fasting plasma glucose (FPG), total cholesterol (TC), triglycerides (TGs), low-density lipoprotein cholesterol, high-density lipoprotein cholesterol, serum sodium, serum potassium, alanine aminotransferase (ALT), and aspartate transaminase (AST) levels were measured.

Patients were followed up primarily through telephone contact. If no response was received, follow-up was terminated at the time of the patient’s last available medical record. The endpoint of this study was all-cause mortality in patients with HF.

FAR was defined as the ratio of the plasma FIB level (g/L) to the plasma ALB level (g/L). C-FAR was calculated as the serum CRP level (mg/L) × FAR. Because the raw values of BNP had a highly skewed distribution, they were logarithmically transformed.

### 2.3 Statistical analysis

We divided the patients into a low C-FAR group (C-FAR <0.69) and a high C-FAR group (C-FAR ≥ 0.69) according to the median C-FAR value. Continuous variables were classified as normally or nonnormally distributed by the normality test. The independent samples *t* test was used to compare differences in normally distributed continuous variables, and the Mann‒Whitney *U* rank sum test was used to compare differences in nonnormally distributed data. Continuous variables are presented as the mean ± standard deviation if normally distributed or as the median with interquartile range if nonnormally distributed. Categorical variables are summarized as numbers and percentages. Between-group differences in categorical variables were compared using the *χ* ^2^ test.

The cumulative incidence of all-cause death was calculated using Kaplan–Meier plots and the log-rank test. The restricted cubic spline models allowed us to determine the association of C-FAR with the risk of all-cause mortality. Unadjusted univariate Cox proportional hazard regression analyses were applied to show the estimated impact of each variable on all-cause mortality. Multivariate analysis using COX regression model tested variables that were significant (*P* < 0.05) in the univariate analysis to determine independent predictors of all-cause mortality. Multivariate Cox proportional hazards models were applied to determine the associations of C-FAR with the incidence rates of all-cause mortality. The first unadjusted group was regarded as the reference group. The results are expressed as hazard ratios (HRs) with 95% confidence intervals (CIs). Time-dependent receiver operating characteristic (ROC) curves and the corresponding area under the curve (AUC) were calculated to compare the predictive ability of C-FAR in HF patients.

The correlation coefficient was calculated for statistical correlations between continuous variables based on Spearman’s nonparametric test. In addition, we performed stratification analysis to confirm whether the effect of FAR and CRP differed in each of the subgroups. Patients with HF were divided into group 1 (FAR < 0.091 + CRP < 7.470), group 2 (FAR < 0.091 + CRP ≥ 7.470), group 3 (FAR ≥ 0.091 + CRP < 7.470), and group 4 (FAR ≥ 0.091 + CRP ≥ 7.470) according to the median FAR and CRP values.

Data were analyzed statistically using SPSS ver. 25.0 and R 4.3.1. A double-sided *P* value <0.05 was considered statistically significant.


**Informed consent:** Informed written consent was obtained from all patients before the intervention.
**Ethical approval:** This study was endorsed by the medical ethics committee of the First Affiliated Hospital of Kunming Medical University and complied with the Declaration of Helsinki.

## 3 Results

### 3.1 Baseline patient characteristics

After excluding patients lost to follow-up and those with missing data, 1,196 HF patients were enrolled in the study. The median age of the patients was 67 years, and 38.0% were women. Compared to the low C-FAR group, patients in the high C-FAR group had higher values of WBC, FIB, CRP, FPG, UA, creatinine, and sodium and worse NYHA class, while they had lower values of RBC, hemoglobin, and GFR and a higher proportion of combined diabetes (all *P* values <0.05) (Table 1).

**Table 1:** Baseline characteristics according to C-FAR

Characteristics	Total (*n* = 1,196)	Low C-FAR group (*n* = 598)	High C-FAR group (*n* = 598)	*P v*alue
Age, years	66.83 ± 12.51	65.55 ± 12.90	68.11 ± 12.00	0.113
Female, *n* (%)	454 (38.0)	239 (40.0)	215 (47.4)	0.153
BMI, kg/m^2^	23.02 ± 3.81	22.98 ± 3.96	23.06 ± 3.66	0.304
NYHA, *n* (%)				0.001
Class Ⅳ	444 (37.1)	194 (32.4)	250 (41.8)	
Heart rate (bpm)	85.24 ± 21.02	83.05 ± 20.08	87.43 ± 21.71	0.023
SBP, mmHg	122.10 ± 22.94	122.20 ± 22.65	122.00 ± 23.26	0.927
DBP, mmHg	76.23 ± 15.04	76.79 ± 15.20	75.67 ± 14.88	0.212
Medical history, *n* (%)				
Smoking status	409 (34.2)	195 (32.6)	214 (35.8)	0.247
Drinking status	201 (16.8)	100 (16.7)	101 (16.9)	0.983
Coronary heart disease	619 (51.8)	298 (49.8)	321 (53.7)	0.183
Hypertension	660 (55.2)	319 (53.3)	341 (57.0)	0.201
Diabetes	341 (28.5)	147 (24.6)	194 (32.4)	0.003
Atrial fibrillation	406 (33.9)	202 (33.8)	204 (34.1)	0.903
LVEF				0.812
HFrEF	499 (41.7)	249 (41.6)	250 (41.8)	
HFmrEF	233 (19.5)	113 (18.9)	120 (20.1)	
HFpEF	464 (38.8)	236 (39.5)	228 (38.1)	
Laboratory data				
WBC (10^9^/L)	6.93 (5.54, 9.07)	6.47 (5.34, 8.21)	7.39 (5.78, 10.09)	<0.001
RBC (10^12^/L)	4.55 ± 0.77	4.61 ± 0.72	4.48 ± 0.80	0.041
PLT (10^9^/L)	192.00 (148.00, 243.00)	192.50 (152.00, 240.00)	191.00 (145.75, 247.25)	0.730
Fibrinogen (g/L)	3.57 ± 1.30	3.12 ± 0.96	4.01 ± 1.43	<0.001
ALB (g/L)	36.69 ± 4.55	37.93 ± 4.23	35.46 ± 4.53	0.465
Hemoglobin (g/L)	138.11 ± 24.17	140.15 ± 22.70	136.06 ± 25.41	0.006
Lg BNP	3.17 ± 0.28	3.13 ± 0.27	3.20 ± 0.29	0.155
CRP (mg/L)	7.47 (3.00, 21.78)	3.00 (1.60, 5.00)	21.69 (12.60,47.89)	<0.001
FPG (mmol/L)	5.03 (4.16, 6.50)	4.89 (4.12, 5.99)	5.12 (4.22, 7.15)	<0.001
UA (μmol/L)	477.10 (370.50, 588.10)	467.90 (365.00, 567.10)	493.35 (382.08, 606.55)	0.016
Creatinine (μmol/L)	103.50 (83.20, 134.10)	98.85 (81.13, 125.35)	108.70 (86.68, 140.90)	<0.001
GFR (ml/min)	44.10 (32.33, 56.69)	46.13 (34.87, 59.36)	42.16 (29.74, 54.13)	<0.001
Sodium (mmol/L)	141.01 ± 4.43	141.55 ± 4.01	140.46 ± 4.75	0.001
Potassium (mmol/L)	3.94 ± 0.60	3.96 ± 0.58	3.92 ± 0.62	0.203
Chlorine (mmol/L)	102.92 ± 4.68	103.62 ± 4.49	102.21 ± 4.76	0.148
Total cholesterol (mmol/L)	3.64 ± 1,01	3.73 ± 0.99	3.56 ± 1.03	0.552
TG (mmol/L)	1.10 (0.86, 1.50	1.10 (0.85, 1.53)	1.10 (0.87, 1.46)	0.768
AST (U/L)	28.60 (20.03, 43.28)	28.20 (20.28,40.00)	28.85 (20.00, 48.93)	0.081
ALT (U/L)	25.05 (16.70, 42.30)	25.00 (16.88, 41.00)	25.80 (16.25, 45.40)	0.513
Medications at admission, *n* (%)				
CRT/ICD	116 (9.7)	54(9.0)	62(10.4)	0.895
SGLT2i	263 (22.0)	145 (24.2)	118 (19.7)	0.059
Beta-blocker	690 (57.7)	365 (61.1)	325 (54.4)	0.615
Aldosterone antagonist	947 (79.2)	435 (72.7)	474 (79.3)	0.943
ACEI/ARB/ARNI	587 (49.1)	330 (55.2)	257 (43.0)	0.321
Diuretics	930 (77.8)	397 (66.4)	533 (89.1)	0.838

BMI: body mass index; NYHA: New York Heart Association; SBP: systolic blood pressure; DBP: diastolic blood pressure; LVEF: left ventricular ejection fraction; WBC: white blood cell; RBC: red blood cell; PLT: blood platelet; ALB: albumin; BNP: B-type natriuretic peptide; CRP: C-reactive protein; FPG: fasting plasma glucose; UA: uric acid; GFR: glomerular filtration rate; AST: alanine aminotransferase; ALT: aspartate transaminase; SGLT2i: sodium-glucose cotransporter 2 inhibitor; ACEI: angiotensin-converting enzyme inhibitor; ARB: angiotensin receptor inhibitor; ARNI: angiotensin receptor neprilysin inhibitor.

Note: low C-FAR: C-FAR < 0.69, high C-FAR: C-FAR ≥ 0.69.

Differences in normally distributed continuous variables were compared using variance analyses, and those in nonnormally distributed data were compared using Mann‒Whitney *U* tests. chi-square tests were used to compare differences in categorical variables between groups.

### 3.2 Prediction of the incidence of all-cause mortality by C-FAR

**Figure 1:**
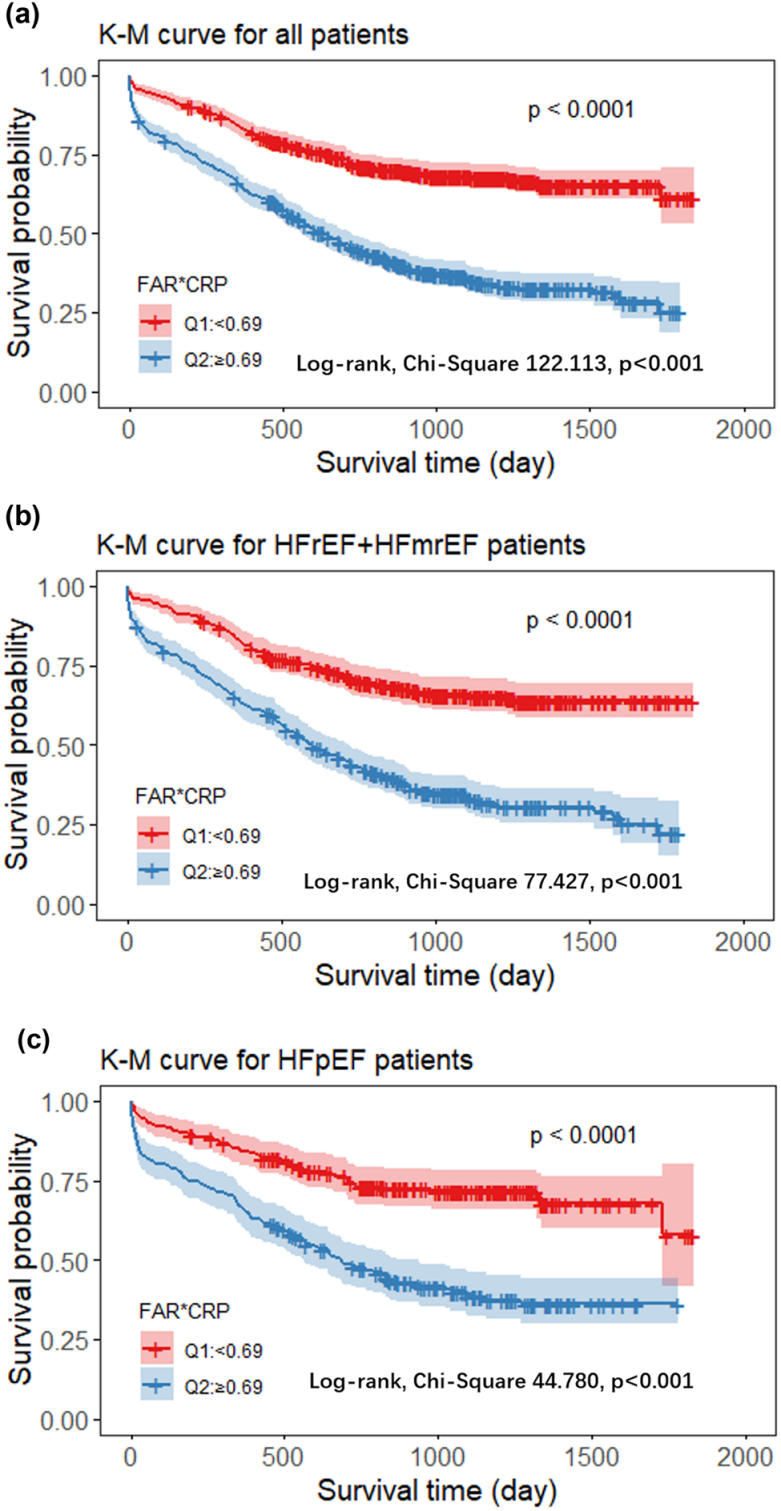
Kaplan‒Meier analysis for all-cause mortality according to different C-FAR levels. (a) All HF patients, (b) HFrEF plus HFmrEF patients, and (c) HFpEF patients. Note: Q1: low C-FAR, C-FAR < 0.69, Q2: high C-FAR, C-FAR ≥ 0.69. The line represents the reference value of the survival rate, and the corresponding color area represents the CI. The all-cause mortality of the high C-FAR group was higher than the low C-FAR group for all patients, regardless of the type of HF.

Kaplan‒Meier analysis showed that the cumulative incidence of all-cause mortality was higher in the high C-FAR group (C-FAR ≥ 0.69) than in the low C-FAR group (C-FAR <0.69) for all patients (log-rank test, chi-square 122.113, *P* < 0.001) (Figure 1a), HFrEF + HFmrEF patients (log-rank test, chi-square 77.427, *P* < 0.001) (Figure 1b), and HFpEF patients (log-rank test, chi-square 44.780, *P* < 0.001) (Figure 1c).

**Figure 2:**
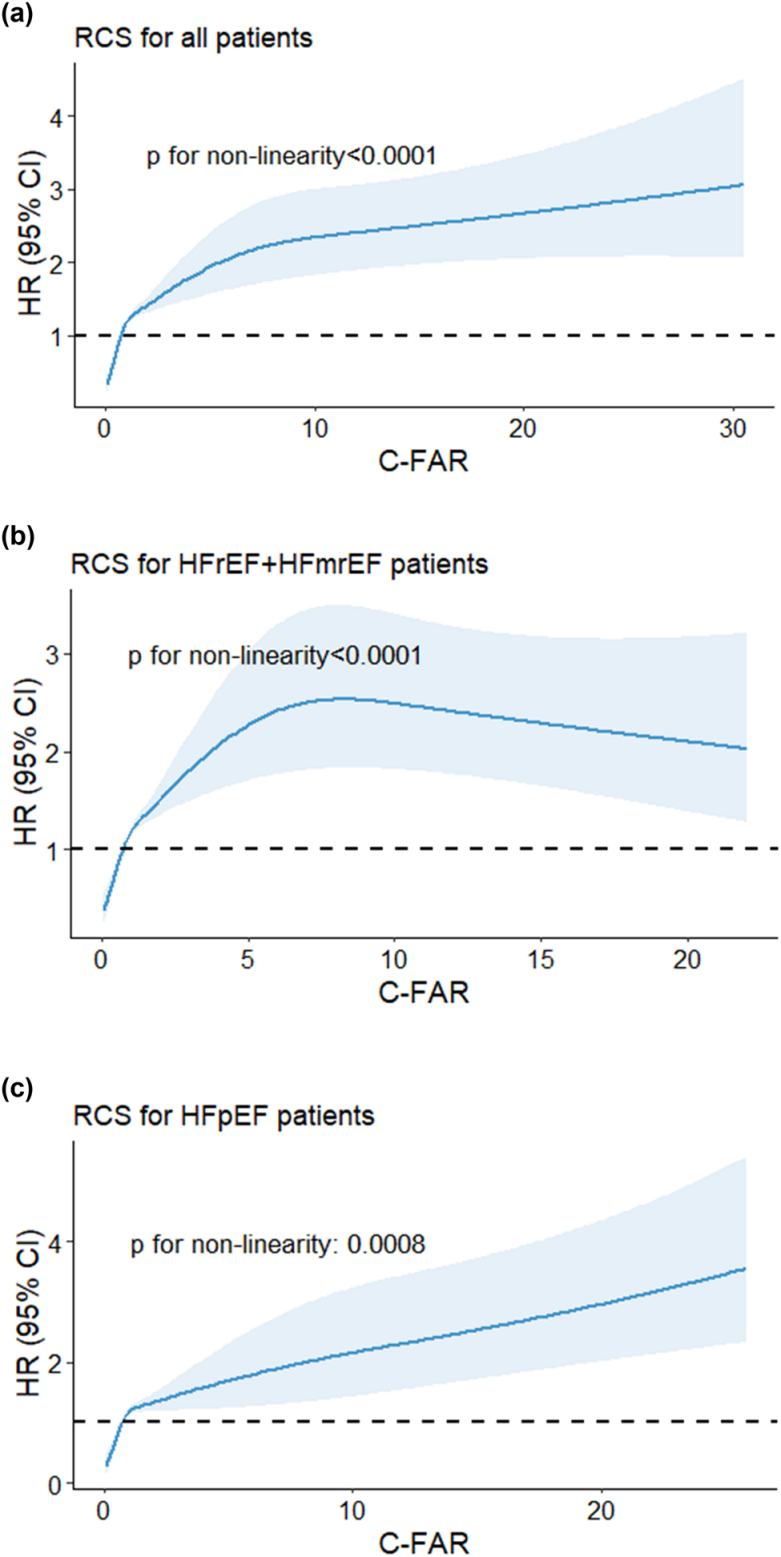
Restricted cubic spline analysis for the association of C-FAR and all-cause mortality. (a) All HF patients, (b) HFrEF plus HFmrEF patients, and (c) HFpEF patients. Note: HRs are indicated by solid lines and 95% CIs by shaded areas.

As shown in Figure 2, we used restricted cubic splines to flexibly model and visualize the relation of C-FAR levels with all-cause mortality in all HF patients and different types of HF patients. C-FAR was roughly positively correlated with the risk rate of all-cause death for all patients. In HFpEF patients, C-FAR was also roughly positively correlated with the risk rate of all-cause death. However, in HFrEF + HFmrEF patients, C-FAR was positively correlated with the risk of all-cause death at first and then negatively correlated.

### 3.3 C-FAR as an independent predictor

Table 2 shows the four multivariate Cox proportional hazard models used to determine the correlation between the C-FAR groups and all-cause mortality. In model 4, the covariates adjusted for included age, body mass index, NYHA cardiac function classification, Lg BNP, creatinine, serum UA, and GFR. With the low C-FAR group as a reference, high C-FAR was associated with a higher incidence of all-cause mortality in all HF patients and in different types of HF patients. Compared with low-level C-FAR patients, the HR of high-level C-FAR patients increased by 1.168 times in all HF patients, by 1.152 times in HFrEF plus HFmrEF patients, and by 0.915 times in HFpEF patients (*P* < 0.001).

**Table 2:** Cox proportional hazards models for the association of C-FAR and the risk of all-cause mortality

Model	All HF patients		HFrEF plus HFmrEF		HFpEF	
	HR (95% CI)	*P*	HR (95% CI)	*P*	HR (95% CI)	*P*
Unadjusted	2.601 (2.181, 3.101)	<0.001	2.582 (2.074,3.216)	<0.001	2.641 (1.965, 3.551)	<0.001
Adjusted model 1	2.464 (2.066, 2.940)	<0.001	2.466 (1.979, 3.073)	<0.001	2.437 (1.812, 3.278)	<0.001
Adjusted model 2	2.512 (2.105, 2.998)	<0.001	2.516 (2.019,3.136)	<0.001	2.479 (1.843, 3.335)	<0.001
Adjusted model 3	2.267 (1.897, 2.709)	<0.001	2.207 (1.757, 2.772)	<0.001	1.996 (1.471, 2.707)	<0.001
Adjusted model 4	2.168 (1.808, 2.600)	<0.001	2.152 (1.710, 2.707)	<0.001	1.915 (1.407, 2.606)	<0.001

Ref.: low C-FAR (C-FAR <0.69).

Adjusted model 1: adjusted for age.

Adjusted model 2: adjusted for age and BMI.

Adjusted model 3: model 2 + NYHA cardiac function classification and Lg BNP.

Adjusted model 4: model 3 + creatinine, serum UA, and GFR.

### 3.4 Predictive ability of C-FAR

**Figure 3:**
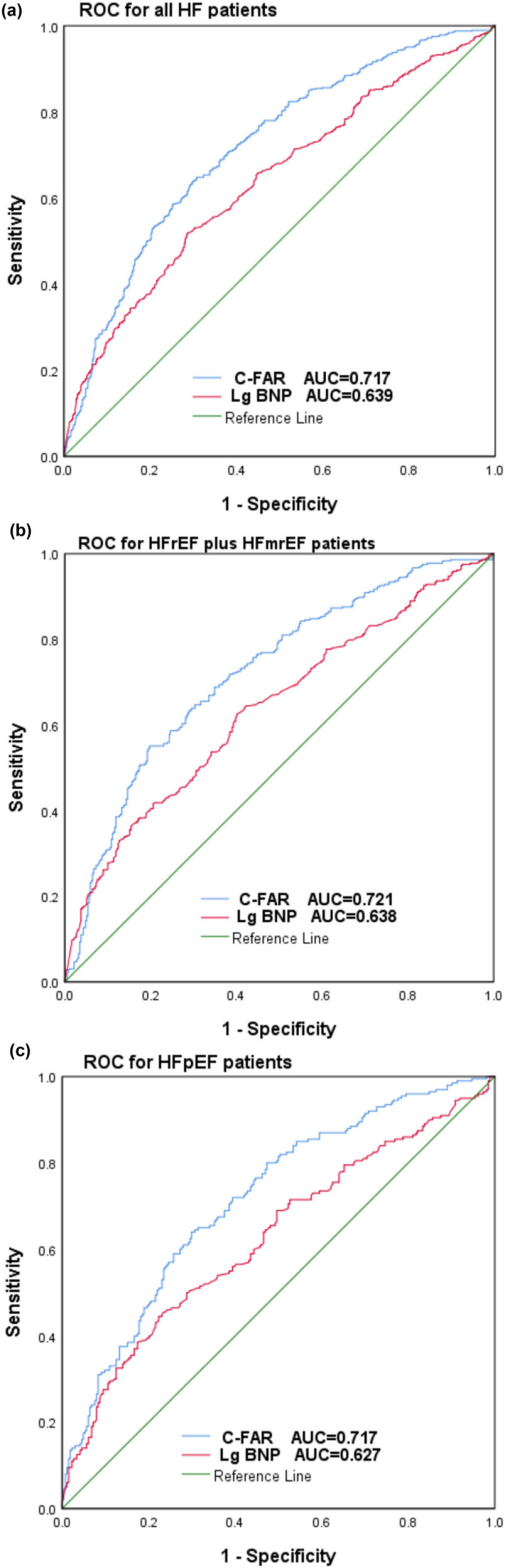
Time-dependent ROC curves of C-FAR with the reference line for all-cause mortality. (a) All HF patients. (b) HFrEF plus HFmrEF patients. (c) HFpEF patients.

We constructed time-dependent ROC curves to investigate the ability of C-FAR to predict all-cause mortality in HF patients. In ROC curve analysis, C-FAR had an AUC of 0.717 (95% CI 0.688–0.746), with a sensitivity of 63.9% and a specificity of 70.0%, for predicting the prognosis of all HF patients. C-FAR was significantly better than Lg BNP (AUC 0.639, 95% CI 0.608–0.671, *P* < 0.001) (Figure 3a).

C-FAR was also better than Lg BNP in predicting the prognosis of patients with different types of HF. Among patients with HFrEF plus HFmrEF, the AUC of C-FAR was 0.721 (95% CI 0.684–0.758) and that of Lg BNP was 0.638 (95% CI 0.598–0.678) (Figure 3b). Among patients with HFrEF, the AUC of C-FAR was 0.717 (95% CI 0.670–0.763) and that of Lg BNP was 0.627 (95% CI 0.575–0.679) (Figure 3c) (all *P* values were <0.001).

### 3.5 Correlation and subgroup analysis

The Spearman’s rank correlation analysis results are reported in Table A1. FAR was positively correlated with CRP in the total participant population [Spearman’s correlation coefficient (*r*): 0.318, *P* < 0.001]. In addition, C-FAR was positively correlated with age, LVEF, Lg BNP, FPG, WBC, PLT, AST, and creatinine but negatively correlated with RBC, HB, serum sodium, serum chlorine, GFR, and TC (*P* < 0.05).

There was an interaction between FAR and CRP, so subgroup analysis was performed to determine the association. All patients with HF were divided into four groups (group 1: FAR < 0.091 + CRP < 7.470, group 2: FAR < 0.091 + CRP ≥ 7.470, group 3: FAR ≥ 0.091 + CRP < 7.470, group 4: FAR ≥ 0.091 + CRP ≥ 7.470). Using group 1 as a reference, group 4 patients had the highest risk of all-cause mortality (Table 3).

**Table 3:** Subgroup analysis

	Unadjusted		Adjusted	
	HR (95% CI)	*P*	HR (95% CI)	*P*
**All HF patients**				
FAR < 0.091	Ref.		Ref.	
FAR ≥ 0.091	1.252 (1.061, 1.478)	0.008	1.195 (1.003, 1.425)	0.046
CRP < 7.470	Ref.		Ref.	
CRP ≥ 7.470	2.900 (2.427, 3.465)	<0.001	2.182 (1.811, 2.629)	<0.001
**Combined categories**				
Group 1: FAR < 0.091 + CRP < 7.470	Ref.		Ref.	
Group 2: FAR < 0.091 + CRP ≥ 7.470	2.468 (1.928, 3.158)	<0.001	1.591 (1.226, 2.066)	<0.001
Group 3: FAR ≥ 0.091 + CRP < 7.470	0.818 (0.602, 1.111)	0.199	0.723 (0.530, 0,988)	0.042
Group 4: FAR ≥ 0.091 + CRP ≥ 7.470	2.827 (2.262, 3.532)	<0.001	2.117 (1.674, 2.675)	<0.001
**HFrEF plus HFmrEF patients**				
FAR < 0.091	Ref.		Ref.	
FAR ≥ 0.091	1.187 (0.996, 1.459)	0.102	1.142 (0.919, 1.419)	0.230
CRP < 7.470	Ref.		Ref.	
CRP ≥ 7.470	2.986 (2.386, 3.736)	<0.001	2.369 (1.870, 3.000)	<0.001
**Combined categories**				
Group 1: FAR < 0.091 + CRP < 7.470	Ref.		Ref.	
Group 2: FAR < 0.091 + CRP ≥ 7.470	2.617 (1.954, 3.504)	<0.001	1.768 (1.291, 2.422)	<0.001
Group 3: FAR ≥ 0.091 + CRP < 7.470	0.728 (0.483, 1.098)	0.130	0.620 (0.408, 0,944)	0.026
Group 4: FAR ≥ 0.091 + CRP ≥ 7.470	2.757 (2.095, 3.626)	<0.001	2.170 (1.628, 2.893)	<0.001
**HFpEF patients**				
FAR < 0.091	Ref.		Ref.	
FAR ≥ 0.091	1.461 (1.093, 1.952)	0.010	1.306 (0.956, 1.784)	0.093
CRP < 7.470	Ref.		Ref.	
CRP ≥ 7.470	2.753 (2.052, 3.693)	<0.001	1.826 (1.332, 2.503)	<0.001
**Combined categories**				
Group 1: FAR < 0.091 + CRP < 7.470	Ref.		Ref.	
Group 2: FAR < 0.091 + CRP ≥ 7.470	2.082 (1.303, 3.328)	0.002	1.215 (0.738, 2.000)	0.445
Group 3: FAR ≥ 0.091 + CRP < 7.470	0.992 (0.617, 1.597)	0.975	0.878 (0.542, 1.424)	0.598
Group 4: FAR ≥ 0.091 + CRP ≥ 7.470	3.107 (2.107, 4.580)	<0.001	2.038 (1.335, 3.109)	0.001

Note: Adjusted for age, BMI, NYHA cardiac function classification, Lg BNP, PLT, HB, chlorine, AST, and UA.

## 4 Discussion

This study suggested that C-FAR plays an important role in predicting the prognosis of patients with different types of HF. Kaplan‒Meier analysis showed that the all-cause mortality of the high C-FAR group was higher regardless of the type of HF. In all four Cox proportional risk models, for all HF patients and for different HF subgroups (HFrEF plus HFmrEF and HFpEF), C-FAR was an independent predictor of all-cause mortality. Subgroup analysis showed that patients had the highest risk of all-cause mortality when FAR ≥ 0.091 and CRP ≥ 7.470. Among all HF patients, the risk of death was 2.117 times higher than that in the FAR < 0.091 and CRP < 7.470 group; among HFrEF plus HFmrEF patients, it was 2.170 times higher; and among HFpEF patients, it was 2.038 times higher. The time-dependent ROC curves showed that the AUC for C-FAR was 0.717 (*P* < 0.001) in all HF patients, with a sensitivity of 63.9% and a specificity of 70.0%, which provided an incremental prognostic value beyond that of plasma BNP (AUC = 0.639). In HFrEF plus HFmrEF and HFpEF patients, C-FAR was also better than Lg BNP in predicting the prognosis of patients.

FIB is a marker of thrombosis and inflammation and is associated with the prognosis of many diseases, including coronary artery disease [20], diabetes [21], and chronic kidney disease [22]. There is growing evidence that FIB is a poor prognostic predictor of CVD [23,24,25]. FIB may increase cardiovascular risk through platelet aggregation, plasma viscosity, and fibrin formation. A study by Kotbi et al. has also shown that FIB is related to the severity of coronary artery disease and cardiovascular risk in Moroccan patients [26]. ALB is a protein synthesized in the liver that influences nutrient absorption, colloidal pressure, and systemic inflammation [27]. In HF, hypoalbuminemia may be a marker of comorbid burden, inflammatory state, malnutrition, and cachexia. Low serum ALB levels are associated with an increased risk of HF onset and progression. Hypoproteinemia may promote pulmonary bruising, myocardial edema, and subsequent worsening of myocardial dysfunction, diuretic resistance, and fluid retention and reduce antioxidant function and anti-inflammatory properties [28,29,30,31]. In patients with HFpEF, the excessive activation of renin–angiotensin–aldosterone system leads to the expansion of blood volume, and the circulating blood volume continues to increase through progressive sodium and water retention, resulting in the decrease of plasma ALB concentration. In patients with HFrEF, to maintain circulatory homeostasis, the sympathetic nervous system and the renin–angiotensin–aldosterone system are continuously activated through continuous efforts [32,33]. In this process, the over-expression of bioactive molecules has toxic effects on the heart and circulation, accompanied by the further activation of inflammatory signaling pathway [34] which leads to the increase of CRP and FIB levels. The role of FAR, as a ratio of FIB to ALB, has been elucidated in several diseases. FAR has been associated with poor prognosis in a variety of cancers [35,36,37,38] and has also been directly associated with the severity of coronary artery calcification and poor prognosis in acute coronary syndromes [15,39,40], but it is less studied in HF. In addition, it is more sensitive and specific in predicting major adverse cardiovascular events than FIB and ALB alone [14].

Serum CRP level has been widely considered to be a nonspecific but sensitive marker of the acute inflammatory response. During acute inflammation, CRP levels can be increased by 100 times or even 500 times. This reaction is mainly regulated by proinflammatory cytokines, especially IL-6 [41]. Many prospective studies have shown that plasma CRP is a strong independent predictor of the risk of acute myocardial infarction, stroke, peripheral arterial disease, and vascular death [42,43,44]. Anand et al. found that higher CRP levels are associated with features of more severe HF and are independently associated with mortality and morbidity [18]. Jin et al. reported that CRP was an excellent prognostic marker for HFrEF, HFmrEF, and HFpEF [45]. The primary cause of elevated CRP is related to cardiac decompensation and ongoing damage to other organs; low cardiac output and venous stasis may induce IL-6 production. This key cytokine activates CRP through TNF-α production, which in turn activates complement and amplifies the inflammatory response; this may lead to myocardial tissue damage or dysfunction [46]. The contribution of CRP to the progression of HF may also be related to effects on organs other than the heart. The common comorbidities of HF, anemia, and renal dysfunction may be caused in part by inflammatory activation [47]. However, the specific inflammatory processes that lead to elevated CRP levels in patients with HF and their specific mechanisms for disease progression have not been clarified.

## 5 Conclusion

This study indicates that FAR and CRP are independent predictors of prognosis in CHF patients. C-FAR was significantly associated with the incidence of all-cause mortality in HF patients, regardless of HF subtype, and roughly positively correlated with the risk of all-cause death. Our study demonstrated that C-FAR can predict the prognosis of patients with HF.

## 6 Limitations

This is a retrospective observational study, and data bias could not be avoided despite correcting for multiple confounding factors. Further prospective studies are needed to validate the role of C-FAR in the prognosis of patients with CHF. The risk of selection bias in retrospective research is inevitable, which might affect the results’ generalizability. Patients with HF who were in NYHA class III or IV were the primary subjects in this study. The predictive value of the C-FAR for all-cause mortality in HF patients with NYHA class I or II was not explored.

## Appendix

**Table A1:** Correlation coefficients between C-FAR and other factors

Variable	FAR		CRP		C-FAR	
	***r***	*p*	*r*	*p*	*r*	*p*
FAR	1		0.318	<0.001		
CRP	0.318	<0.001	1			
Age	0.057	0.047	0.065	0.025	0.064	0.026
BMI	0.019	0.505	−0.001	0.985	0.025	0.391
LVEF	0.145	<0.001	0.029	0.323	0.071	0.014
NYHA	0.012	0.676	0.112	<0.001	0.132	<0.001
Lg BNP	−0.004	0.885	0.093	0.001	0.087	0.003
FPG	0.135	<0.001	0.117	<0.001	0.137	<0.001
WBC	0.193	<0.001	0.277	<0.001	0.277	<0.001
RBC	−0.181	<0.001	0.094	0.001	−0.106	<0.001
PLT	0.288	<0.001	0.008	0.784	0.071	0.013
HB	−0.212	<0.001	−0.092	0.001	−0.114	<0.001
Sodium	−0.111	<0.001	−0.127	<0.001	−0.111	<0.001
Potassium	−0.014	0.641	0.001	0.976	−0.010	0.741
Chlorine	−0.061	0.035	−0.150	<0.001	−0.131	<0.001
ALT	−0.076	<0.001	0.110	<0.001	0.038	0.190
AST	−0.022	0.443	0.132	<0.001	0.088	0.002
Creatinine	0.161	<0.001	0.111	<0.001	0.097	0.001
UA	−0.112	<0.001	0.060	0.040	0.058	0.048
GFR	−0.094	0.001	−0.109	<0.001	−0.091	0.002
TC	0.021	0.472	−0.127	<0.001	−0.114	<0.001

FAR: fibrinogen-to-albumin ratio; CRP: C-reactive protein; BMI: body mass index; LVEF: left ventricular ejection fraction; NYHA: New York Heart Association; BNP: B-type natriuretic peptide; FPG: fasting plasma glucose; WBC: white blood cell; RBC: red blood cell; PLT: blood platelet; HB: haemoglobin; ALT: aspartate transaminase; AST: alanine aminotransferase; UA: uric acid; GFR: glomerular filtration rate.
